# Modeling of the metallic port in breast tissue expanders for photon radiotherapy

**DOI:** 10.1002/acm2.12320

**Published:** 2018-03-30

**Authors:** Jihyung Yoon, Yibo Xie, David Heins, Rui Zhang

**Affiliations:** ^1^ Physics and Astronomy Louisiana State University Baton Rouge LA USA; ^2^ Mary Bird Perkins Cancer Center Baton Rouge LA USA

**Keywords:** metallic port, modeling, postmastectomy radiotherapy, tissue expander, treatment planning system

## Abstract

The purpose of this study was to model the metallic port in breast tissue expanders and to improve the accuracy of dose calculations in a commercial photon treatment planning system (TPS). The density of the model was determined by comparing TPS calculations and ion chamber (IC) measurements. The model was further validated and compared with two widely used clinical models by using a simplified anthropomorphic phantom and thermoluminescent dosimeters (TLD) measurements. Dose perturbations and target coverage for a single postmastectomy radiotherapy (PMRT) patient were also evaluated. The dimensions of the metallic port model were determined to be 1.75 cm in diameter and 5 mm in thickness. The density of the port was adjusted to be 7.5 g/cm^3^ which minimized the differences between IC measurements and TPS calculations. Using the simplified anthropomorphic phantom, we found the TPS calculated point doses based on the new model were in agreement with TLD measurements within 5.0% and were more accurate than doses calculated based on the clinical models. Based on the photon treatment plans for a real patient, we found that the metallic port has a negligible dosimetric impact on chest wall, while the port introduced significant dose shadow in skin area. The current clinical port models either overestimate or underestimate the attenuation from the metallic port, and the dose perturbation depends on the plan and the model in a complex way. TPS calculations based on our model of the metallic port showed good agreement with measurements for all cases. This new model could improve the accuracy of dose calculations for PMRT patients who have temporary tissue expanders implanted during radiotherapy and could potentially reduce the risk of complications after the treatment.

## 1 INTRODUCTION

More and more postmastectomy patients have immediate breast reconstructions mainly for cosmetic reasons.^1^, ^2^, ^3^, ^4^, ^5^, ^6^, ^7^ A temporary tissue expander, which usually includes a high‐density magnetic injection port, offers many advantages including relative simplicity, low morbidity, and good aesthetic results, over other types of breast reconstructions.^3^, ^6^ American Society of Plastic Surgeons reported that there were 74,694 breast reconstructions using tissue expander/implant in 2014 in the US and the number kept increasing.^8^

Many patients will receive postmastectomy radiotherapy (PMRT) with the temporary tissue expander present, while the tissue expander could negatively impact the effectiveness of PMRT and increase the risk of complications.^9^, ^10^ Some physicians felt the reconstructions challenged their ability to deliver effective radiotherapy.^6^ It has also been reported that failures in the breast reconstruction and complication rates were significantly higher for patients who received PMRT with the temporary tissue expanders than patients who received PMRT with permanent implants^11^ or autologous tissue reconstruction.^12^, ^13^

Previous literature about the effect of the tissue expander containing the high‐density metallic injection port on dose distribution was conflicting and controversial: Moni et al.^14^ used thermoluminescent dosimeters (TLD) and found no significant component of scatter dose around the metallic port, no increased dose at the surface of the expander, and no excess dose due to the metallic port in the expander; Thompson and Morgan^15^ used diode dosimetry in a water phantom and reported the attenuation of up to 30% of local dose for a single beam, and treatment target could be underdosed by approximately 10% in clinical situations using tangential parallel opposed beams. They also concluded the modeling of this dose perturbation in treatment planning systems (TPS) was inadequate; Damast et al.^16^ reported that the potential dose perturbation of the tissue expander could be as much as 22% for a single 6 MV beam and 16% for a single 15 MV beam based on ex vivo film dosimetry. The in vivo film measurement one side at a time for one PMRT patient treated with 15 MV beams concurred with ex vivo results, while TLD measurements for six patients treated with 15 MV beams showed smaller dose variation (86%~101% of prescription dose). They recommended using 15 MV photons with compensating bolus to treat patients; Chatzigiannis et al.^17^ used Monte Carlo (MC) simulation and found 7% to 13% dose reduction with the expander in place for two 6 MV tangential photon beams, and around 6% dose reduction for 18 MV beams; Chen et al.^18^ reported that tissue expanders with metal ports will increase dose heterogeneity and reduce dose coverage significantly for patients treated with 6 MV or 18 MV opposed tangent photon fields; Sharabi et al. reported in an abstract^19^ 5%~20% dose attenuation due to the metallic port and claimed a nondeformable implant model of the port was created based on manufacture specifications, but they did not show the details of the model or validate the dosimetric accuracy of the model; Trombetta et al.^20^ initially reported that no significant change in dose distributions was found for an opposed pair of 6 MV photon beams delivered to a breast phantom containing a metallic port, but later drew a conflicting conclusion in a separate paper^21^ that the metallic port must be taken into account in the dose calculations; Strang et al.^22^ used TLD measurements and concluded that radiation doses around the tissue expander were unaltered; Srivastava et al.^23^ conducted measurements in a water phantom using a small ion chamber (IC) and concluded that dose perturbation caused by metallic port in photon beams was 5%~20% and this perturbation could not be predicted by TPS; Zabihzadeh et al.^24^ used MC simulation and found a dose enhancement about 15% in front of the port and a dose reduction of about 10% at 5 cm under the port; Gee et al.^25^ used radiochromic films as in vivo dosimeter and found an average 7% dose reduction to skin surface in a sample of PMRT patients with the tissue expander present during radiotherapy.

One of the reasons for these contradictory findings is that most of the current TPSs are not calibrated or validated for the high‐density metallic port in the tissue expander. Considering TPS is an essential step among the whole radiotherapy procedure, it is critical to calibrate it to generate accurate treatment plans for patients with tissue expanders. The modeling of the metallic port in the current TPS may not be able to accurately calculate the dosimetry impact introduced by the high‐density materials, particularly in the areas near these materials.^16^, ^18^ Thompson and Morgan^15^ manually assigned a bulk density of 7.9 g/cm^3^ to the implant in TMS TPS (Nucletron, Veenendaal, the Netherlands) and concluded this kind of modeling was ineffective; Chen et al.^18^ used a series of phantom and film measurements to find an electron density relative to water of 11.8 for the metallic port in an open field photon beam in Eclipse TPS (Varian Medical Systems, Palo Alto, CA, USA). However, this value did not yield good agreement between measurements and TPS calculations for both 6 and 18 MV photon beams; Trombetta et al.^20^ assigned the density of the metallic port in the Eclipse TPS to be 5 g/cm^3^ which is the highest available value in the system. Except for one abstract^19^ in which the details and accuracy of the model were not provided, none of the previous studies created and validated a model of the metallic port with generic dimensions and density that can be applied to different beam energies, neither did they investigate the impact of the metallic port on any advanced techniques like intensity‐modulated radiotherapy (IMRT) or volumetric‐modulated arc therapy (VMAT), while these techniques have been used to treat postmastectomy patients.^26^

In this study, we aim to model the metallic port in a commercial TPS (Pinnacle version 9.8 TPS, Philips Healthcare, Fitchburg, WI, USA) based on radiological properties of the port. We optimized our model until TPS calculations matched measurement results, and validated the model using a simplified anthropomorphic phantom. We also compared the new model with two widely used clinical models in the phantom and for a patient case.

## 2 MATERIAL AND METHODS

### 2.1 Modeling of the metallic port

**Figure 1:**
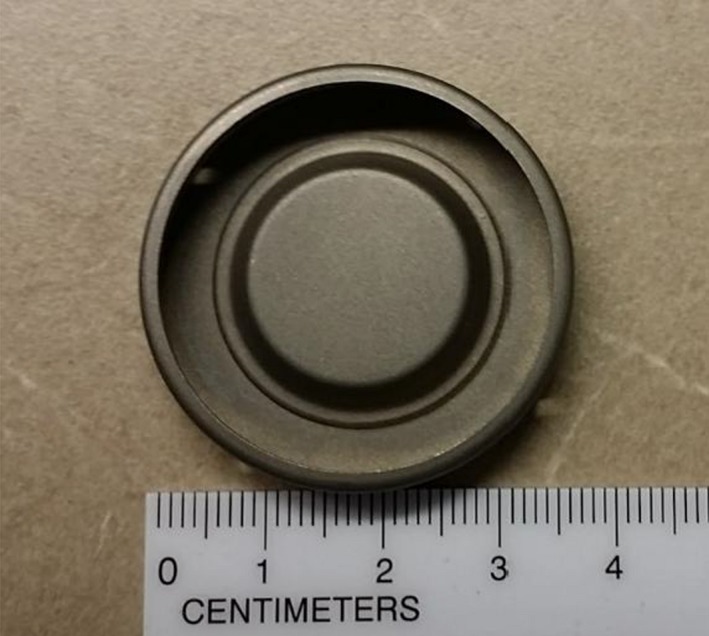
Image of a typical metallic injection port taken out of a breast tissue expander (ALLERGAN, Santa Barbara, CA).

A metallic port (Fig. 1) was separated from the MAGNA‐SITE^®^ injection site in the Natrelle^®^ 133 Tissue Expander (ALLERGAN, Santa Barbara, CA) for this study. The metallic port consists of a magnetic disk (Nd_2_Fe_14_B; Neodymium magnet, nominal density = 7.4 g/cm^3^) with physical dimensions of 2.1 cm diameter and 3.5 mm thickness, and a Titanium shell casing (nominal density = 4.2 g/cm^3^) with physical dimensions of 3.5 cm diameter and 0.4 mm thickness.

To model the dimensions of the metallic port in the Pinnacle version 9.8 TPS, we measured cross‐sectional profiles of transmitted beams through the metallic port by film instead of using its physical dimensions. A piece of Gafchromic EBT3 film (Ashland, Bridgewater, NJ, USA) was placed on the bottom of a water tank at 100 source‐to‐axis distance (SAD) without any back‐scattering phantom to reduce scatter contribution. The metallic port was placed on the film surface parallel or perpendicular to the radiation beam and the water tank was filled with 5 cm depth of water. A 6 MV photon beam with 10 × 10 cm^2^ field was delivered using an Elekta VersaHD™ linac (Elekta Corporation, Stockholm, Sweden). The irradiated films were scanned with an Epson Expression 10000XL (Seiko Epson Corporation, Nagano, Japan). Since the attenuation by surrounding Ti casing was small, the full width a half maximum (FWHM) of the magnetic disk was measured to determine the dimension of the metallic port model.

**Figure 2:**
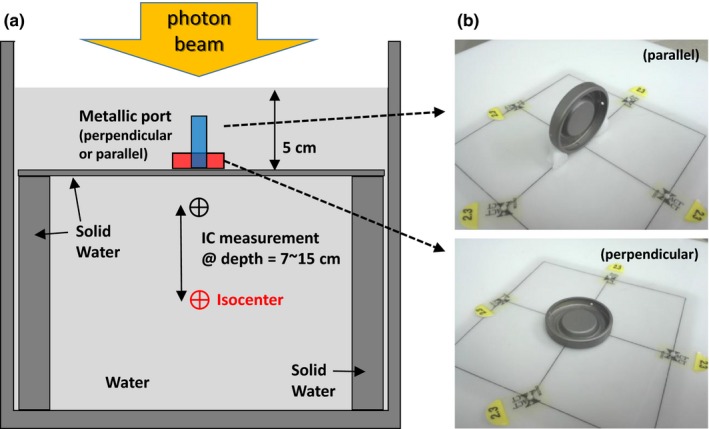
(a) Schematic illustration of the ion chamber (IC) measurement setup. The IC was placed directly under the metallic port and the distance between the IC and water surface varied between 7 cm and 15 cm. (b) The metallic port was placed on a solid water phantom surface with parallel (top) and perpendicular (bottom) setup.

The density of the metallic port was determined by comparing TPS calculations with IC (31006 PTW Pinpoint Ionization Chamber, effective volume 0.015 cm^3^, PTW, Freiburg, Germany) measurements. The measurement setup is shown in Fig. 2: the isocenter was located at 15 cm depth and the source‐to‐surface (SSD) was 85 cm; the metallic port was put on the surface of a solid water slab and the distance from the water surface to the slab surface was 5 cm. The IC was placed directly under the metallic port to measure the dose attenuation through the metallic port and the position of the IC varied from 7 to 15 cm under the water surface. To confirm if the IC was exactly aligned with the metallic port for the parallel setup, the location of the metallic port was slightly adjusted laterally until the IC reading reached the minimum. The measured doses at depths were compared with dose calculations by collapsed cone convolution (CCC) algorithm in Pinnacle TPS with a dose grid of 1 × 1 × 1 mm^3^. Although there are more accurate dose algorithms 27 that can be used to reduce metal artifact and calculate dose around high‐density heterogeneities, the CCC algorithm was chosen because it is the default and also the most accurate dose algorithm used by Pinnacle and the purpose of this study was to calibrate the Pinnacle TPS to accurately calculate dose around the metallic port. The density in the TPS model of the metallic port was adjusted with an extended CT conversion table in Pinnacle until the calculation results agreed with the measurements. However, the attenuation from the metallic port model is determined not only by the density of the model but also the dimensions (both diameter and thickness) of the model in TPS. For example, increasing the diameter of the metallic port model in the TPS will yield more attenuation in the parallel direction [Fig. 2(b)], while the effect in the perpendicular direction would not be significant. Also, increasing the density of the model will yield increased dose close to the metallic port because of increased scatter, while doses at deeper points will decrease due to increased attenuation. Therefore, modeling of the metallic port in the TPS requires fine adjustments of the combination of density, diameter, and thickness. In this study, we aimed to find the best model that yields the best agreement between all TPS calculations and measurements. As a result, the dimension of the metallic port model may be different from the film measurement result.

**Figure 3:**
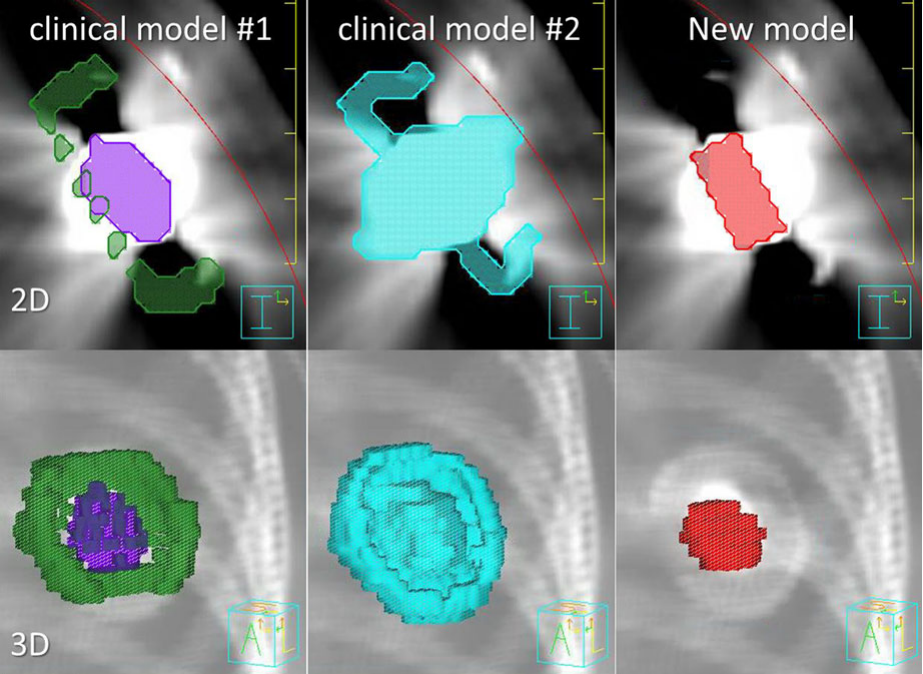
Different metallic port models used in this study. 2D and 3D images of each model are shown.

Contouring the metallic port may have large uncertainties because of the presence of artifacts induced by the metallic port in the kilovoltage (kV) CT images, and most patients only have kV CT images available. In some clinic, part of the artifact is included in the metallic port contouring which can cause possible dose error. In this study, we compared our new model with two widely used clinical models (Fig. 3): in clinical model #1, the titanium shell and magnetic disk were contoured by the dosimetrist based on the physical dimensions, CT images, and “bone” window/level. The densities of the Titanium shell and magnetic disk were overridden by their nominal densities, and surrounding artifacts were overridden as water; in clinical model #2, the metallic port was contoured based on CT images only with “bone” window/level and some of the artifacts were therefore included. The surrounding artifacts were overridden as water and the contoured metallic port utilized the assigned default density converted from the CT number; in our new model, the location of the disk and its tilted angles in transverse and sagittal plane were identified from the CT images using “bone” window/level. The geometrical information of the metallic port, such as the location of the center of the disk and tilted angles in lateral and sagittal plane on the CT images, was transferred to an in‐house MATLAB (Mathworks, version 7.9, Natick, MA, USA) code to model the disk three‐dimensionally. We then generated the contour of the metallic port using the in‐house code and used the new contour to update the file containing the structure set in Pinnacle. The density of the metallic port was overridden by the value determined from IC measurement described previously. The surrounding artifacts were overridden as water.

### 2.2 Validation of the new metallic port model and two clinical models

To validate our model in a more clinically realistic situation, simplified anthropomorphic phantoms were used to simulate a patient body. Because different physicians prefer tissue expanders with various amount of fluid inside during radiation,^6^ we simulated both completely deflated and inflated implant cases to evaluate the accuracy of our model in these two extreme situations. A 6.3‐cm water equivalent solid block was placed on a wooden lung phantom to simulate a fully inflated tissue expander. Under the block, a 1‐cm Superflab bolus was place on the lung phantom to simulate the chest wall. For a deflated tissue expander, a 2‐cm block was used to represent the tissue expander. The whole phantom was scanned by a GE LightSpeed 16 Slice computed tomography (CT) scanner (GE Healthcare, Little Chalfont, UK) and the CT images with 2.5 mm slice thickness were imported into Pinnacle 9.8 TPS.

**Figure 4:**
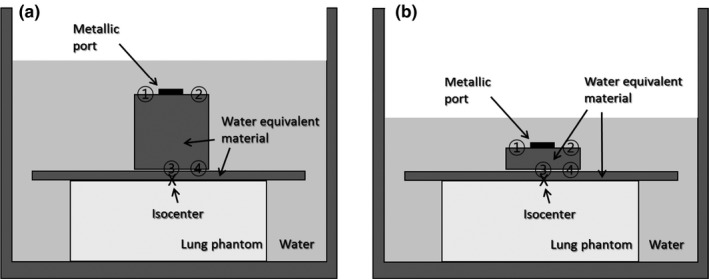
Schematic illustration of TLD measurement setup in simplified anthropomorphic phantoms simulating (a) inflated and (b) deflated tissue expanders implanted in a patient's body. Numbers with circle represent TLD measurement points.

Thermoluminescent dosimeters were placed to measure the dose around the metallic port as shown in Fig. 4, and the total number of TLDs was 28 (24 plus 1 background and 3 calibration TLDs). The SSD was 90.7 cm for inflated case and 95 cm for deflated case, and the distance between metallic port and water surface was 2 cm for both cases. Measurement points 1, 2, and 4 were located 2.5 cm away from the center of the port, and point 3 was directly under the metallic port. A two‐field open field (gantry angles 90° and 270°) plan with a field size of 10 × 10 cm^2^ at isocenter, a volumetric‐modulated arc therapy (VMAT) plan (a single 180° arc from 90° to 270°) and a four‐field IMRT (4fld‐IMRT) (gantry angles 70°, 90°, 270°, and 290°) plan were generated for the phantom. For all plans, the isocenter was located at the interface between lung and chest wall and was aligned with the center of the metallic port, as shown in Fig. 4. For 4fld‐IMRT and VMAT plan optimizations, the lung phantom and an imaginary planning target volume (PTV) which included the measurement points were contoured in the Pinnacle TPS. A single fraction of 1 Gy was prescribed. TPS calculated point doses based on the new model and two clinical models were compared with TLD measurements.

After TLD measurements, we performed TLD calibrations by sandwiching a TLD packet in solid water phantoms, delivered a known dose to the TLD packet and recorded the TLD reading. This was repeated for several dose levels and a calibration curve was created based on the readings. The TLD packets were read using a REXON UL‐320 Reader (Rexon Components, Inc., Beachwood, OH, USA). The TLD heating curve lasts 30 s and contains two plateaus, one 50° and one 240°. Each TLD packet (i.e., measurement point) was filled with approximate 45 mg of TLD powder. The TLD powder in each packet was divided into three samples of approximately 15 mg each and the three samples were used to determine the mean dose and standard deviation of the mean for each TLD packet. To minimize daily variation due to decay after irradiation, TLD reading were actually started after 2 days so that signal fading after irradiation could be ignored.

The possible TLD uncertainties include fading, dose–response nonlinearity, energy response corrections, and system sensitivity. Among these uncertainties, the fading effect was basically negligible because TLD reading were started after 2 days so that signal decay after irradiation could be ignored; energy response corrections were not necessary because we used the same beam energy for both measurements and calibrations; the dose–response nonlinearity was calibrated during TLD calibrations by delivering several known dose levels (the dose range covers the expected measured dose values) to the reference TLDs and doing a linear least‐squares fit of the data. The fitting is usually very good and it has a much smaller variance than the other factors according to Kirby et al.^28^; the system sensitivity of our TLD reader was well established and the average standard error of the mean dose was well within 4%.^29^

### 2.3 Comparing the new model with clinical models for a patient case

**Figure 5:**
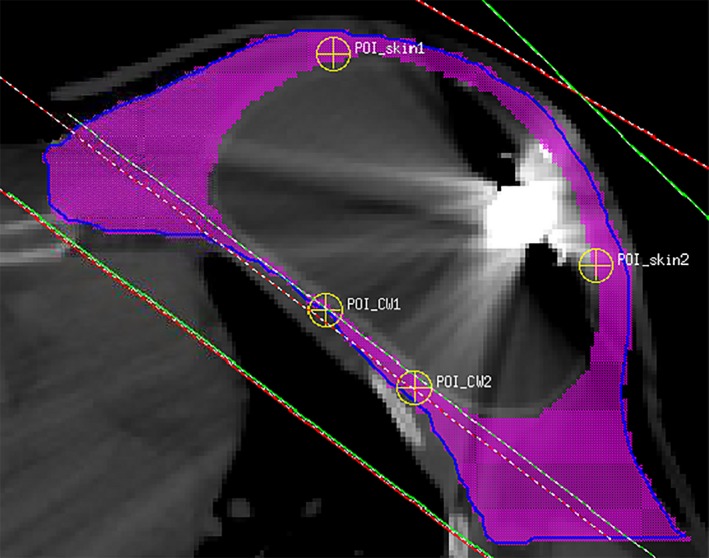
Photon dose calculation points in a PMRT patient with a tissue expander. The blue contour represents PTV, and the purple color wash represents reduced‐PTV (PTV minus tissue expander) on this slice.

To further compare the new model with the clinical models, point doses and dose–volume histograms (DVHs) were calculated for a patient case. A conventional plan containing a pair of opposite tangent beams with wedges, a VMAT plan and a 4fld‐IMRT plan were tested. The PTV included the chest wall, supraclavicular area, axillary area, and internal mammary chain area. The dose prescription was 50 Gy administered in 25 fractions for all the plans. For the conventional plan, a 6 MV open beam with gantry angle 307° together with a 20° wedge, and a 10 MV open beam with gantry angle 127° together with a 21° wedge were used. For the VMAT plan, a dual‐arc with 220° rotations was used to cover the PTV. The beam geometry consisted of a 0° couch angle and a 45° collimator angle. The 4fld‐IMRT plan consisted of three 6 MV IMRT beams with gantry angles of 324°, 304°, and 124°, and one 10 MV IMRT beam with gantry angle of 160° to cover the whole PTV. For comparison, a baseline condition (no disk) was created with the metallic port and the tissue expander overridden as water to simulate a homogeneous breast without a tissue expander. Doses at two points close to the skin but at depths deeper than 1 cm and doses at two points on the chest wall were calculated (Fig. 5). A “reduced‐PTV” was defined for the DVH evaluation purpose: the original PTV is narrowed down to the slices containing the metallic port in the CT images and the tissue expander plus the metallic port were excluded because we were only interested in the dose delivered to the patient's tissue.

## 3 RESULTS & DISCUSSION

**Figure 6:**
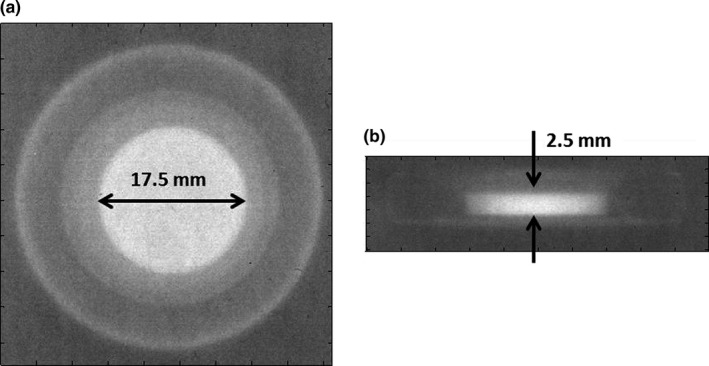
Projection images of the metallic port on Gafchromic films for (a) perpendicular and (b) parallel setup.

The film measurement results for parallel and perpendicular setups are shown in Fig. 6. It was found that the radiological diameter of the metallic port was 1.75 cm and the thickness was 2.5 mm, and these were smaller than the nominal diameter (2.1 cm) and thickness (3.5 mm) that included Ti shell. Ti casing caused a very dim shadow in the film image. This was expected, since the effective thickness of the Ti wall was less than 0.2 g/cm^2^ (= 4.5 g/cm^3^ × 0.04 cm), which can be ignored without noticeable change in the calculated doses.

**Figure 7:**
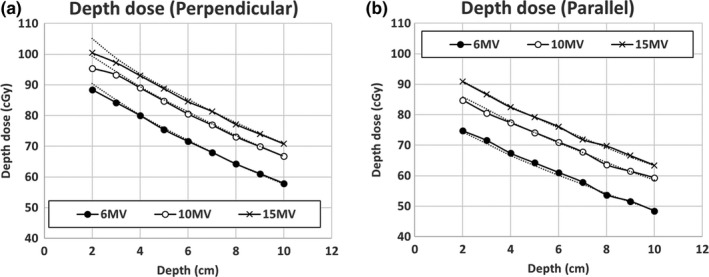
Depth doses under the metallic port for (a) perpendicular and (b) parallel setup for 6, 10, and 15 MV photons with field size of 10 × 10 cm^2^. (Solid lines) Ion chamber measurement and (dotted lines) TPS calculations were plotted on the same graph. Note the depth here means the distance between the metallic port and ion chamber.

Using the determined dimensions from the film measurements, we found that the calculated doses in the TPS did not match the IC measurements with the perpendicular setup when the density was adjusted for the parallel setup measurements. To achieve the best agreement for both setups, the thickness and the diameter of the metallic port were decided to be 5 mm and 1.75 cm, and the density of the port was decided to be 7.5 g/cm^3^. The IC measurements and calculated doses are shown in Fig. 7, and they show good agreement (within 1%) for all depths and photon energies except at 2 cm depth with the perpendicular setup where the difference was 4.1% for 10 MV photons and 4.7 % for 15 MV photons. This could be attributed to the fact that the radiation beam was significantly attenuated by the metallic port with the perpendicular setup, while TPS could not handle this high density heterogeneity satisfactorily. Because the metallic port is very thin, the side scatter in water smeared out this dose drop at shallow depth with the parallel setup. However, since the thickness of a typical tissue expander is greater than 2 cm, this dose difference would not affect the accuracy of dose calculation within a patient's tissue.

The calculated and measured doses using the simplified anthropomorphic phantoms are shown in Table 1 together with the discrepancies between measured and calculated doses. Large discrepancy was expected at locations where the dose impact of the metallic port and the limitation of TPS's capability of handling high density heterogeneity were manifest. For the inflated case, and especially for open and 4fld‐IMRT plans, dose perturbations caused by the metallic port were significant at points 1 and 2 because both plans contained beams that were parallel to the port, while points 3 and 4 were relatively further away from the metallic port. This was also supported by the fact that the dose discrepancies at points 3 and 4 were almost identical for different port models. For VMAT plans, the dose discrepancies were more uniformly distributed because of the characteristics of VMAT beams (rotational). The dose discrepancies between TPS calculations and TLD measurements were larger when clinical model 1 or 2 was used, while our new model introduced smaller discrepancies in most cases. For the deflated case, all four points were closer to the metallic port and the largest discrepancy showed up at different locations for different models. The dose discrepancies were overall larger than those in the inflated case, especially for clinical model 1 or 2, because less scatter doses were generated in the smaller volume of water surrounding the metallic port and these scatter doses could smear out the dose impact of the metallic port. The new model still provided much better accuracy in most cases. Overall, the TPS calculations based on the two clinical models showed worse agreement with TLD measurements compared with the new model. For all the measurement points, TPS calculations based on the new model agreed with TLD measured doses within 5.0%, which was within the accuracy limit of TLD. Therefore, the new photon metallic port model was validated for different beams in the phantoms.

**Table 1:** TLD measurements (mean ± standard deviation of the mean) and TPS calculated doses based on different models for open beam, VMAT, and 4fld‐IMRT plans for simplified anthropomorphic phantoms. TLD measurements were used as the reference for dose difference calculations. (TE: tissue expander)

TE status	Plan	Point	TLD	Clinical #1		Clinical #2		New	
			Dose (cGy)	Dose (cGy)	Difference (%)	Dose (cGy)	Difference (%)	Dose (cGy)	Difference (%)
Inflated	Open	1	94.8 ± 1.4	86.7	−8.5	96.5	1.8	94.2	−0.6
		2	93.6 ± 1.8	88.8	−5.2	97.1	3.7	95.9	2.4
		3	91.9 ± 1.1	91.4	−0.5	91.4	−0.5	91.4	−0.5
		4	92.8 ± 2.7	93.4	0.6	93.4	0.6	93.4	0.6
	VMAT	1	99.9 ± 2.4	97.7	−2.2	98.6	−1.3	98.8	−1.1
		2	95.3 ± 0.9	94.0	−1.3	96.0	0.8	95.6	0.3
		3	91.0 ± 0.9	90.3	−0.8	90.7	−0.4	90.6	−0.5
		4	93.7 ± 1.8	90.9	−3.0	91.2	−2.7	91.3	−2.6
	4fld‐IMRT	1	106.9 ± 0.1	100.2	−6.3	102.8	−3.9	104.0	−2.7
		2	104.1 ± 1.0	96.0	−7.8	98.0	−5.8	99.3	−4.6
		3	97.2 ± 0.4	99.5	2.4	99.5	2.4	99.5	2.4
		4	93.8 ± 1.9	98.5	5.0	98.5	5.0	98.5	5.0
Deflated	Open	1	95.7 ± 1.8	85.3	−10.8	103.5	8.2	93.8	−2.0
		2	99.1 ± 1.7	89.1	−10.0	104.7	5.7	97.6	−1.5
		3	97.3 ± 2.1	96.1	−1.2	103.5	6.4	96.4	−0.9
		4	96.6 ± 0.8	97.7	1.1	105.3	9.0	98.0	1.4
	VMAT	1	103.8 ± 2.1	98.2	−5.4	109.9	5.9	100.4	−3.3
		2	103.0 ± 2.3	97.8	−5.1	108.7	5.5	99.2	−3.7
		3	96.8 ± 0.6	92.6	−4.3	102.4	5.8	95.0	−1.8
		4	100.7 ± 1.2	94.4	−6.2	105.7	5.0	97.8	−2.9
	4fld‐IMRT	1	98.2 ± 3.5	89.3	−9.0	104.0	5.9	95.7	−2.5
		2	99.0 ± 1.7	88.8	−10.3	102.1	3.1	95.6	−3.4
		3	104.0 ± 1.1	100.4	−3.4	107.7	3.6	99.9	−3.9
		4	100.6 ± 2.2	101.2	0.6	108.2	7.6	100.8	0.2

Point doses within the PMRT patient are listed in Table 2. For all plans and models, the dosimetric impact of the metallic port on chest wall was negligible, while the port introduced significant dose shadows in skin area, especially in the conventional plan. For the VMAT and 4fld‐IMRT plans, the effect of the metallic port was less significant compared with the conventional plan due to the increased number of beam angles. Compared with the new model, clinical model #1 overestimated the dose attenuation from the metallic port, while clinical model #2 underestimated the attenuation.

**Table 2:** Calculated doses based on different models for conventional, VMAT, and 4fld‐IMRT plans for a PMRT patient. The “no disk” was used as the reference for dose difference calculations. (CW: chest wall)

Plan	Point location	No disk	Clinical 1		Clinical 2		New	
		Dose (cGy)	Dose (cGy)	Difference (%)	Dose (cGy)	Difference (%)	Dose (cGy)	Difference (%)
Conventional	CW1	4562.6	4564.9	0.05	4563.2	0.01	4563.5	0.02
	CW2	4561.8	4566.5	0.10	4563	0.03	4563.1	0.03
	skin1	5336.7	4815.8	−9.76	5161.4	−3.28	4985.8	−6.58
	skin2	5213.7	4813	−7.69	5046.6	−3.21	4928.3	−5.47
VMAT	CW1	4972.2	4968.3	−0.08	4967	−0.10	4951.5	−0.42
	CW2	4887.8	4872	−0.32	4875.8	−0.25	4857.4	−0.62
	skin1	5090.2	5050.3	−0.78	5070	−0.40	5050.9	−0.77
	skin2	5250.2	4892.8	−6.81	5099.1	−2.88	4988.6	−4.98
4fld‐IMRT	CW1	4972.4	4971.3	−0.02	4973.2	0.02	4973.7	0.03
	CW2	5021.9	5020.8	−0.02	5024	0.04	5024.8	0.06
	skin1	5312	4986.7	−6.12	5183.5	−2.42	5119.4	−3.63
	skin2	5533.9	5205.9	−5.93	5378.7	−2.80	5333	−3.63

**Figure 8:**
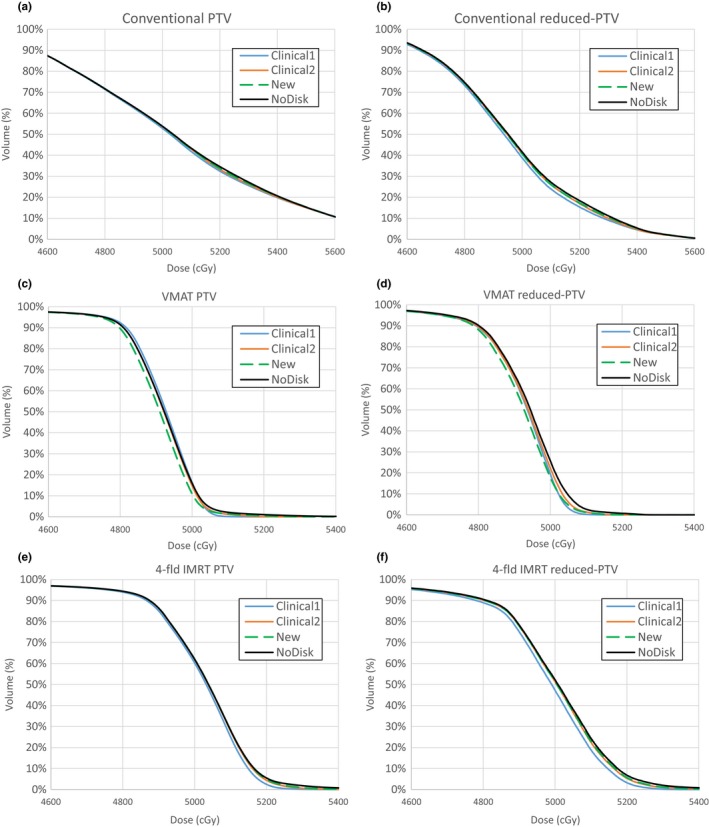
DVHs for PTV and reduced‐PTV for (a–b) conventional, (c–d) VMAT, and (e–f) 4fld‐IMRT plans for a patient case.

The DVHs for the PTV and reduced‐PTV are shown in Fig. 8. Overall, the choice of the metallic port model has a small impact on the whole PTV DVH because the portion of the volume of the metallic port in the PTV was small, except VMAT plan in which the DVH curve was shifted to the left with the new model. For the reduced‐PTV, the choice of the model has a more pronounced effect and the shift of the DVH curves depends on the plan and the models in a complex way. The change in target coverage caused by the metallic port was less significant than that reported by Chen et al.,^18^ and it is possibly due to the different models and TPSs used in their study and ours. Since point dose calculations revealed that the metallic port has a negligible impact on chest wall, the DVHs for organs at risk like lung or heart were not analyzed because they are further away from the port than chest wall.

Our results are consistent with previous studies. Gee et al.^25^ reported an average 7% dose reduction to skin surface in a sample of PMRT patients with tissue expanders using opposing tangent beams, and Chatzigiannis et al.^17^ reported 7% to 13% dose reduction with the expander in place for two 6 MV tangential photon beams, and our study showed 5.5%~6.6% dose reduction in the skin area for the conventional plan. The dose reduction increased to 15.6%~15.8% when we only used one beam in the conventional plan, which is close to 22% reduction reported by Damast et al.^16^ for a single 6 MV beam.

The strength of this study is that a generic and simplified model of the metallic port for all possible conditions was developed and validated. Because of the simple geometry, the contours of the model can be reproduced easily or be stored as a template in Pinnacle. The model can be used for any photon treatment and a short script in Pinnacle can be used to add the metallic port contour to patients' plans automatically. The only things that need to be adjusted in the script for a specific patient are the center position and the orientation of the model which can be quantified by checking the planning CT images. The density of the metallic port should be overridden by the value determined in this study (7.5 g/cm^3^), and the surrounding artifacts should be overridden as water. Our research will allow TPS to accurately calculate dose distribution surrounding the metallic port. Without this information, clinicians may ignore the dose perturbation or prescribe an inaccurate amount of additional dose to compensate for the miscalculated dose shadow, which may either cause the loss of target coverage or increase the risk of complications like capsular contracture since radiation to the breast after reconstruction can significantly increase the rates of these complications.^10^, ^30^ The methodology utilized in this study can also be used to investigate other high‐density materials within the patient, e.g., metal implants within chordoma patients, pacemaker, hip prosthesis, dental implants, etc.

One of the limitations of this study is that we only investigated one type of the tissue expander (McGhan Style 133, ALLERGAN, Santa Barbara, CA, USA), while other brands of tissue expanders are also used for breast reconstruction,^20^ and only modeled the metallic port in one TPS (Pinnacle, Philips Healthcare, Fitchburg, WI, USA). However, it seems that the tissue expander used in this study was the most popular type used in the US and other countries according to literature^14^, ^15^, ^16^, ^17^, ^24^, ^25^, ^31^ and Pinnacle is one of the most widely used TPS worldwide, which means our model is applicable to most clinics. The methodology used in this study is generally applicable and clinics that use different brands of tissue expanders or TPS could create their own model and improve their dosimetric accuracy by using our approach. Second, only one patient's plans were used for clinical evaluations. As we mentioned in the introduction section, our goal is to model the metallic port and we already validated and compared the new model with the clinical models using simplified anthropomorphic phantoms. The patient case was used as an example to further demonstrate the necessity of using our new model to calculate doses and to show the dose perturbation depends on the plan and the models.

## 4 CONCLUSIONS

We have modeled the metallic port within the tissue expanders in a commercial TPS based on radiological measurements. Calculation results from the new model agreed with IC measurements within 1% for most cases. Using simplified anthropomorphic phantoms, the TPS calculated point doses based on the new model agreed with TLD measurements within 5.0% and showed better accuracy than dose calculated based on the clinical models. For a PMRT patient case, we found that the metallic port has a negligible dosimetric impact on chest wall, while the port introduced significant dose shadows in skin area. The current clinical port models overestimate or underestimate the dose perturbation, and as a result, may deliver unexpected local dose to the patient. Therefore, using our model in treatment planning could improve the accuracy of dose delivery for PMRT patients who have temporary tissue expanders implanted during radiotherapy.

## ACKNOWLEDGMENTS

This work was partially supported by National Institutes of Health (NIH) through a National Cancer Institute (NCI) grant K22CA204464, Louisiana State University (LSU) Faculty Research Grant and LSU Economic Development Assistantship Award.

## CONFLICT OF INTEREST

The authors declare no conflict of interest.
